# Blood product transfusion in emergency department patients: a case-control study of practice patterns and impact on outcome

**DOI:** 10.1186/s12245-017-0133-z

**Published:** 2017-02-02

**Authors:** Alexander Beyer, Ryan Rees, Christopher Palmer, Brian T. Wessman, Brian M. Fuller

**Affiliations:** 1 Department of Emergency Medicine, University of Michigan, Ann Arbor, MI 48109 USA; 2 Washington University School of Medicine in St. Louis, St. Louis, MO 63110 USA; 3 Departments of Emergency Medicine and Anesthesiology, Division of Critical Care, Washington University School of Medicine in St. Louis, St. Louis, MO 63110 USA

**Keywords:** Emergency department, Blood transfusion, ARDS

## Abstract

**Background:**

Blood product transfusion occurs in a significant percentage of intensive care unit (ICU) patients. Pulmonary complications, such as acute respiratory distress syndrome (ARDS), occurring in the setting of transfusion, are associated with increased morbidity and mortality. Contrary to the ICU setting, there is little evidence describing the epidemiology of transfusion in the emergency department (ED) or its potential impact on outcome. The objectives of this study were to: (1) characterize transfusion practices in the ED with respect to patient characteristics and pre-transfusion laboratory values; and (2) investigate the effect of ED blood product transfusion on the incidence of pulmonary complications after admission. We hypothesized that blood product transfusion would increase the event rate for pulmonary complications, and have a negative impact on other clinically significant outcomes.

**Methods:**

This was a retrospective case-control study with one-one matching of 204 transfused ED patients to 204 non-transfused controls. The primary outcome was a composite pulmonary outcome that included: acute respiratory failure, new need for ICU admission, and ARDS. Multivariable logistic regression was used to evaluate the primary outcome as a function of transfusion.

**Results:**

One-hundred twenty four (60.8%) patients were transfused packed red blood cells (PRBC) in the ED. The mean pre-transfusion hemoglobin level was 8.5 g/dl. There were 73 patients with a hemoglobin value ≥10 g/dl; 19 (26.0%) received a PRBC transfusion. A total of 54 (26.5%) patients were transfused platelets. The main indications were thrombocytopenia (27.8%) and neurologic injury (24.1%). Ten patients had a platelet level <10,000 (guideline recommended threshold for transfusion to prevent spontaneous hemorrhage). The mean platelet count for neurologic injury patients was 197,000 prior to transfusion. The primary outcome occurred in 26 control patients (12.7%), as compared with 28 cases (13.7%). In multivariable logistic regression analysis, ED transfusion was not associated with an increased odds of primary outcome [adjusted OR 0.91 (0.48–1.72), *P* = 0.77]. The mortality rate was 10.8% in the cases and 8.8% in the controls, *P* = 0.51.

**Conclusions:**

A significant percentage of ED blood product transfusions are discordant with guideline recommendations. However, there was no association with ED transfusion and worse clinical outcome.

**Electronic supplementary material:**

The online version of this article (doi:10.1186/s12245-017-0133-z) contains supplementary material, which is available to authorized users.

## Background

In a general intensive care unit (ICU) population, 20–50% of patients are transfused blood products during their ICU stay [1]. The previous two decades have seen intense investigation into transfusion practices, and their impact on outcome in the ICU [2–4]. This has led to a well-defined epidemiology of transfusion practices in the ICU, and evidence-based guideline recommendations in the critical care setting [5]. The emergency department (ED) is the entry point for the majority of patients in the ICU. Excluding massive transfusion protocols in the setting of major trauma, there is little evidence describing transfusion in the ED setting or its potential impact on outcome.

Blood product transfusion is associated with well-documented risks. From a pulmonary perspective, this includes an association with acute respiratory distress syndrome (ARDS), transfusion-related acute lung injury (TRALI), and transfusion-associated circulatory overload (TACO) [6–10]. Development of these pulmonary complications is associated with an increase in morbidity and mortality. As there is increased interest in prevention of pulmonary complications (such as ARDS) after ICU admission, describing the potential impact that ED-based transfusion has, could be an important step in improving outcome [11].

The objectives of this study were to: (1) characterize the transfusion practices in the ED with respect to patient characteristics and pre-transfusion laboratory values; and (2) investigate the effect of ED blood product transfusion on the incidence of pulmonary complications after admission. We hypothesized that blood product transfusion would increase the event rate for pulmonary complications, and have a negative impact on other clinically significant clinical outcomes.

## Methods

### Study design

This was a retrospective case-control study and is reported in accordance with The Strengthening the Reporting of Observational Studies in Epidemiology (STROBE) Statement: Guidelines for Reporting Observational Studies [12]. The funding organizations played no role in the conduct and reporting of the study. Ethics approval was obtained from the Human Research Protection Office at the corresponding author’s institution with waiver of informed consent.

### Study setting and population

This study was conducted at a university-affiliated, urban teaching hospital (1250 beds), with an annual ED census of >95,000 patients. Over a 4-year period (June 2009 to May 2013), adult patients (age ≥18 years) presenting to the ED were electronically screened for inclusion. Exclusion criteria were: (1) multi-system trauma; (2) discharge from the ED; (3) hemoglobin <7 g/dl; (4) transfer to another hospital from the ED; and (5) death in the ED. The study population was restricted to those patients with a hemoglobin level ≥7 g/dl, as guideline recommendations suggest considering a transfusion below this threshold. Multi-system trauma patients were also excluded, as little controversy exists regarding transfusion in the setting of major hemorrhage.

### Matching

All adult patients admitted to the ED were identified as having received blood product transfusion (cases) in the ED by electronic registry query and verified by review of the medical record. Blood products were defined as packed red blood cells (PRBCs), fresh frozen plasma (FFP), or platelets. Using the same exclusion criteria, along with an electronic screen to identify patients with similar presenting diagnoses, non-transfused patients (controls) that were admitted to the ED over the same time period were identified. The a priori matching strategy was designed based on the assumption that the decision to transfuse blood products in the ED would be based upon the presence of a bleeding condition, laboratory values (i.e., hemoglobin), and age. Therefore, patients were matched one-to-one for key indicators for transfusion: ED diagnosis, hemoglobin value, age, and gender. The matching criteria were: diagnosis (same), hemoglobin (±1 g/dl), age (±5 years), and gender (same) in this order. Non-matched cases were discarded.

### Measurements and key outcome measures

Baseline demographics, comorbid conditions, vital signs at ED presentation, laboratory values, illness severity, ED length of stay, and ED diagnosis were collected from the electronic medical record. Definitions of comorbid conditions are provided in Additional file 1. Sepsis was defined as previously described [13]. Process of care variables in the ED included intravenous fluid, endotracheal intubation, central venous and arterial catheter placement, antibiotics, and vasopressor infusion. To ensure uniform data collection and accuracy, all variables were defined prior to data extraction and placed in a standardized format during the data collection process. Regular meetings and monitoring of data collection were performed, with verification of data accuracy and cross-checking of all data with electronic medical records.

After admission, blood products transfused during the first 24 h were collected. Fluid balance was recorded daily over the first 3 days. Patients were followed until hospital discharge or death.

The primary outcome was a composite pulmonary outcome that included: acute respiratory failure, new need for ICU admission, and the presence of ARDS. This outcome was chosen a priori as it accounted for potential complications and clinical deterioration associated with transfusion occurring both in patients not initially requiring ICU admission, and those critically ill during ED presentation. The primary outcome was restricted to the first 3 days to better examine a temporal link between ED transfusion and complications, including lung injury at 72 h (i.e., “delayed TRALI syndrome”) [14]. Acute respiratory failure was defined as the need for invasive or non-invasive ventilation in patients not initially requiring positive pressure support in the ED. New need for ICU admission was defined as the need for ICU admission in patients initially admitted from the ED to the general ward. ARDS was defined according to the Berlin definition and adjudicated, by co-investigators blinded to transfusion status, as previously described [15–17].

Secondary outcomes included ventilator-, hospital-, and ICU-free days, the need for renal replacement therapy (RRT), as well as hospital mortality.

### Data analysis

Descriptive statistics, including mean [standard deviation (SD)], median [interquartile range (IQR)], and frequency distributions were used to assess the characteristics of the patient cohort. Continuous and categorical variables were compared using an unpaired *t* test, Mann-Whitney *U* test, Chi-square test, or Fisher’s exact test, as appropriate. To assess predictors of the primary outcome, covariates associated with the outcome at *P* < 0.10 were candidates for inclusion in a bidirectional stepwise, multivariable logistic regression analysis. The stepwise regression method selected variables for inclusion or exclusion from the model in a sequential fashion based on the significance level of 0.10 for entry and 0.10 for removal. Collinearity was assessed, and the model used variables that contributed information that was statistically independent of the other variables in the model. The Hosmer-Lemeshow test, along with the examination of residuals, was used to assess model goodness of fit. Adjusted odds ratios (aORs) and corresponding 95% confidence intervals (CI) are reported for variables in the multivariable model, adjusted for all variables in the model.

The expected event rate for the primary outcome in the cases was 20% [3, 8, 16, 18–20]. We estimated that with a sample of 204 patients per group, the study would have 80% power to detect an absolute reduction of 5%, with a two-sided type I error rate of 5%.

## Results

### Study population

**Fig. 1:**
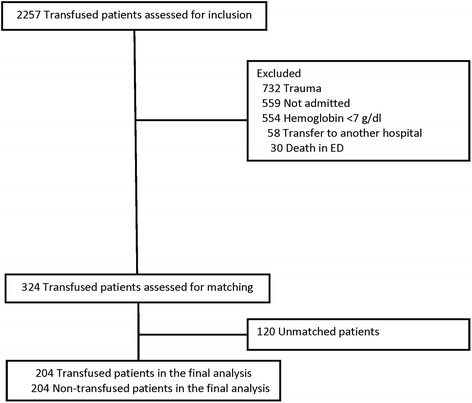
Flow diagram

A total of 2257 transfused patients were assessed for inclusion. A total of 204 matched pairs were included in the final study population (Fig. 1).

Baseline characteristics of the study population are shown in Table 1. The matching strategy yielded a study cohort with similarities in age, gender, ED diagnosis, and hemoglobin values. With respect to other baseline characteristics, in the transfusion group, there was a higher incidence of cirrhosis, along with higher values for INR and bilirubin. With respect to process of care variables, the transfusion group received approximately 600 mL more of intravenous fluid (*P* < 0.001), had a higher incidence of central venous catheter use in the ED (20.1 vs. 7.4%, *P* < 0.001), and were admitted directly to the ICU from the ED more frequently (41.2 vs. 24.5%, *P* < 0.001).

**Table 1:** Baseline characteristics

	Controls: no transfusion in ED group (*n* = 204)	Cases: transfusion group (*n* = 204)	*P* value
Matching criteria			
Age (years)	62.4 (14.9)	62.0 (15.3)	0.78
Male, *n* (%)	103 (50.5)	96 (47.1)	0.49
ED diagnosis, *n* (%)			
Gastrointestinal hemorrhage	49 (24.0)	45 (22.1)	0.64
Infection	44 (21.6)	39 (19.1)	0.54
Cardiac^a^	20 (9.8)	17 (8.3)	0.61
Neurological injury	20 (9.8)	25 (12.3)	0.43
Anemia	20 (9.8)	22 (10.8)	0.74
Metabolic^b^	11 (5.4)	13 (6.4)	0.67
Emergency surgery	10 (4.9)	13 (6.4)	0.52
Liver disease	8 (3.9)	7 (3.4)	0.79
Thrombocytopenia	7 (3.4)	8 (3.9)	0.79
Hypotension	6 (2.9)	9 (4.4)	0.43
Hemorrhage (other)	5 (2.5)	4 (2.0)	0.74
Excessive anticoagulation	1 (0.5)	1 (0.5)	1.0
Other	3 (1.5)	1 (0.5)	0.31
Hemoglobin	9.6 (2.1)	9.5 (2.3)	0.87
Other baseline characteristics			
Race, *n* (%)			
Caucasian	105 (51.5)	114 (55.9)	0.37
African-American	96 (47.1)	83 (40.7)	0.19
Other	3 (1.5)	7 (3.4)	0.20
Comorbidities, *n* (%)			
Diabetes	83 (40.7)	69 (33.8)	0.15
Cirrhosis	27 (13.2)	50 (24.5)	0.004
CHF	42 (20.6)	29 (14.2)	0.09
Dialysis	21 (10.3)	8 (3.9)	0.01
Malignancy	66 (32.4)	69 (33.8)	0.75
COPD	30 (14.7)	28 (13.7)	0.78
Immunosuppression	36 (17.6)	47 (23.0)	0.18
Alcohol abuse	29 (14.2)	24 (11.8)	0.46
Emergency surgery	5 (2.5)	3 (1.5)	0.48
Height (in)	66.5 (4.2)	66.7 (4.5)	0.62
Weight (kg)	81.9 (27.9)	78.5 (26.2)	0.21
PBW (kg)	62.6 (11.1)	63.3 (12.0)	0.57
BMI	28.7 (9.3)	27.4 (9.2)	0.14
Temperature (celsius)	36.8 (0.7)	36.8 (0.8)	0.43
RR	18.6 (3.1)	18.4 (2.8)	0.68
SBP	129.1 (33.6)	122.7 (34.1)	0.06
DBP	72.5 (17.6)	71.4 (17.6)	0.53
Lactate (*n* = 161)	1.5 (1.0–2.7)	2.1 (1.2–3.6)	0.04
Creatinine	1.3 (0.8–2.4)	1.1 (0.8–1.6	0.01
WBC	10.2 (8.4)	10.1 (6.6)	0.88
Platelet	213.6 (146.7)	192.7 (146.5)	0.15
INR	1.6 (1.1)	2.1 (1.7)	0.001
Total bilirubin	0.5 (0.3–0.9)	0.7 (0.4–1.4)	0.001
Albumin	3.3 (0.7)	3.2 (0.8)	0.14
SOFA^c^	4.0 (2.9)	3.6 (2.7)	0.25
Source of admission, *n* (%)			
Home	151 (74.0)	140 (68.6)	0.23
Transferring facility	35 (17.2)	41 (20.1)	0.45
Nursing home	18 (8.8)	23 (11.3)	0.41
Sepsis, *n* (%)	64 (31.4)	73 (35.8)	0.35
ED LOS (hours)	7.1 (5.2–9.7)	8.3 (6.0–11.9)	0.01
Process of care variables			
Intravenous fluids in ED (liters)	1.2 (1.5)	1.8 (1.6)	<0.001
Intubated in ED, *n* (%)	15 (7.4)	24 (11.8)	0.13
Tidal volume, mL/kg PBW	7.3 (6.4–9.0)	8.5 (7.7–10.2)	0.11
Central venous catheter, *n* (%)	15 (7.4)	41 (20.1)	<0.001
Arterial catheter, *n* (%)	8 (3.9)	8 (3.9)	1.0
Antibiotics, *n* (%)	78 (38.2)	85 (41.7)	0.48
Vasopressor infusion, *n* (%)	16 (7.8)	23 (11.3)	0.24
Admitted to ICU, *n* (%)	50 (24.5)	84 (41.2)	<0.001

*ED* emergency department, *CHF* congestive heart failure, *COPD* chronic obstructive pulmonary disease, *PBW* predicted body weight, *BMI* body mass index, *RR* respiratory rate, *SBP* systolic blood pressure, *DBP* diastolic blood pressure, *WBC* white blood cell, *INR* international normalized ratio, *SOFA* sequential organ failure assessment score, *LOS* length of stay, *ICU* intensive care unit

Continuous variables are reported as mean (standard deviation) and median (interquartile range)

^a^Includes the diagnoses of sudden cardiac arrest, heart failure, syncope, acute coronary syndrome, and arrhythmia

^b^Includes the diagnoses of rhabdomyolysis, acute kidney injury, hypoglycemia, diabetic ketoacidosis, and electrolyte abnormalities

^c^Modified score, which excludes Glasgow Coma Scale

### Transfusion characteristics

A total of 124 (60.8%) patients were transfused PRBCs in the ED (Table 2). A mean of 1.9 (±0.8) units per patient was transfused. The most common indications for transfusion were hemorrhage (29.0%), infection (18.5%), anemia (16.9%), and cardiac (9.7%). The mean pre-transfusion hemoglobin level was 8.5 g/dl. There were 73 patients with an initial hemoglobin value ≥10 g/dl; 19 (26.0%) received a PRBC transfusion. Sixty-four (31.4%) patients were transfused FFP in the ED. A mean of 2.2 (±0.9) units per patient was transfused. The most common indications for transfusion were hemorrhage (23.4%), neurologic injury (20.3%), infection (12.5%), and emergency surgery (10.9%). The mean international normalized ratio (INR) prior to transfusion was 3.3. A total of 54 (26.5%) patients were transfused platelets in the ED. A mean of 1.4 (±0.5) units per patient was transfused. The most common indications were thrombocytopenia (27.8%), neurologic injury (24.1%), infection (18.5%), and hemorrhage (14.8%). Of the patients transfused for thrombocytopenia ten (66.7%) had a platelet level <10,000 (guideline recommended threshold for transfusion to prevent spontaneous hemorrhage). The mean platelet count for the neurologic injury patients was 197,000 prior to transfusion.

**Table 2:** Transfusion variables for the 204 patients transfused in the emergency department

	Blood product		
	Packed red blood cells	Fresh frozen plasma	Platelets
Number (%)	124 (60.8)	64 (31.4)	54 (26.5)
Mean (SD)	1.9 (0.8)	2.2 (0.9)	1.4 (0.5)
Indication for transfusion, *n* (%)	Hemorrhage, 36 (29.0) Infection, 23 (18.5) Anemia, 21 (16.9) Cardiac, 12 (9.7)	Hemorrhage, 15 (23.4) Neurologic injury, 13 (20.3) Infection, 8 (12.5) Emergency surgery, 7 (10.9)	Thrombocytopenia, 15 (27.8) Neurologic injury, 13 (24.1) Infection, 10 (18.5) Hemorrhage, 8 (14.8)
Hemoglobin (g/dl)	8.5 (1.7)	10.4 (2.5)	10.7 (2.3)
INR	1.8 (1.4)	3.3 (2.3)	1.5 (1.0)
Platelet	223 (155)	184 (117)	82 (94)

Cardiac: includes the diagnoses of sudden cardiac arrest, heart failure, syncope, acute coronary syndrome, and arrhythmia

During the first 24 h after ED admission, cases were transfused PRBC less frequently compared to controls (23.5 vs. 43.1%, *P* < 0.001); there was a higher incidence of FFP transfusion among the cases (11.8 vs. 5.4% *P* = 0.02). There was no difference in the incidence of platelet transfusion between the cases and controls (10.3 vs. 6.4%, *P* = 0.15) after admission.

### Fluid balance after admission

**Fig. 2:**
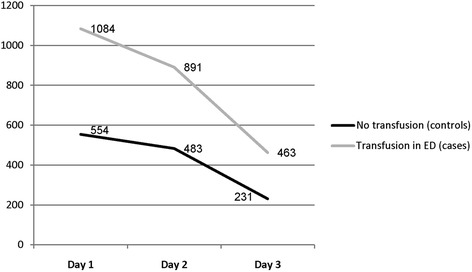
Fluid balance (mL) during the first 3 days of admission. There was a significant difference in net fluid balance during the first 3 days of admission, controls 1.1 l (3.5) vs. 2.1 l (4.5) in the cases, *P* = 0.01

There was a significant difference in net fluid balance during the first 3 days of admission, control group 1.1 l (±3.5) vs. 2.1 l (±4.5) in the cases, *P* = 0.01, Fig. 2.

### Outcomes

The primary outcome occurred in 26 control patients (12.7%), as compared with 28 cases (13.7%). In multivariable logistic regression analysis, adjusting for body mass index, sepsis, shock (i.e., vasopressor use), ED mechanical ventilation, and fluid balance, ED transfusion was not associated with an increased odds of primary outcome [adjusted OR 0.91 (0.48–1.72), *P* = 0.77; Table 3].

**Table 3:** Primary and secondary outcomes according to study group

Outcome	Controls: no transfusion in ED group (*n* = 204)	Cases: transfusion group (*n* = 204)	Odds ratio or between-group difference (95% CI)	*P* value*
Primary composite outcome, *n* (%)	26 (12.7)	28 (13.7)	0.91 (0.48–1.72)	0.77
• Respiratory failure	17 (8.3)	19 (9.3)	1.13 (0.57–2.24)	0.73
• ICU admission	8 (3.9)	7 (3.4)	0.87 (0.31–2.45)	0.79
• ARDS	9 (4.4)	14 (6.9)	1.60 (0.68–3.78)	0.28
Secondary outcomes (days)				
Ventilator-free	24.7 (9.4)	23.7 (10.3)	1.0 (−0.9–2.9)	0.30
ICU-free	24.7 (8.3)	23.7 (9.0)	0.9 (−0.8–2.6)	0.30
Hospital-free	20.0 (8.5)	18.6 (9.1)	1.4 (−0.3–3.1)	0.11
RRT, *n* (%)	18 (8.8)	7 (3.4)	0.37 (0.15–0.90)	0.02
Mortality, *n* (%)	18 (8.8)	22 (10.8)	1.25 (0.65–2.41)	0.51

The primary outcome was a composite outcome that combined the event rate for respiratory failure, ICU admission, and acute respiratory distress syndrome

*P value for the primary outcome measure was a Wald test estimated using a logistic regression model adjusting for body mass index, sepsis, shock (i.e., vasopressor use), ED mechanical ventilation, and fluid balance

*P values for the secondary outcomes are from the Chi-square test (categorical data) and the independent sample *t* test (continuous data)

*CI* confidence interval, *ICU* intensive care unit, *ARDS* acute respiratory distress syndrome, *RRT* renal replacement therapy

Ventilator-, ICU-, and hospital-free days were approximately 1 day higher in the control group; this did not reach statistical significance. The incidence of RRT was 8.8% in the controls and 3.4% in the cases, *P* = 0.02. There was no difference in the mortality rate between the two groups.

There were 70 patients transfused in both the ED and during the first 24 h after admission. The primary outcome occurred in 16 (22.9%) of these patients, as compared with 38 (11.2%) patients that did not receive blood product at both time points [OR 2.34 (1.22–4.49), *P* = 0.009]. There was no mortality difference, 14.3 vs. 8.9%, *P* = 0.17.

## Discussion

On the strength of a number of randomized trials and observational studies in the critical care and peri-operative setting, several guidelines regarding blood product transfusion have been published [5, 21, 22]. The most evidence-based, and physiologically sound, indications for blood product transfusion are for the treatment of life-threatening hemorrhage or coagulopathy, prevention of hemorrhage in the peri-operative/procedural setting, and anemia with evidence of tissue hypoperfusion. Transfusion in the ED could be beneficial if it serves to: (1) improve early hemostasis, resulting in less overall transfusion requirements; or (2) reverse early tissue hypoperfusion, resulting in less subsequent organ failure. It could also be harmful if it promotes transfusion-related complications. However, there is a paucity of data from the ED regarding both transfusion practices and the potential impact on outcome. Results of this case-control study provide some initial data in this domain.

The most common transfusion indication for PRBCs was hemorrhage (primarily gastrointestinal) and infection. The mean hemoglobin level of 8.5 mg/dl is fairly consistent with multicenter observational studies in general ICU patients, however a 26% transfusion rate for patients with a hemoglobin ≥10 g/dl suggests discordance between ED transfusion practices and guideline recommendations [1]. Another significant finding was the frequency of platelet transfusion in the setting of neurologic injury (24.1% of platelet transfusions). A mean platelet count of 197,000 in these patients suggests transfusion was driven by a history of anti-platelet therapy, which is a common practice in our center. The majority of evidence does not support this practice [23, 24].

With respect to clinical outcomes, there was no significant difference between the two groups, contrary to both our a priori hypothesis and the majority of previous data showing an association of harm with transfusion in the critically ill patient. There are several possible explanations. Transfusion therapy is likely safer, owing to improved blood preparation and leukocyte depletion. Our results are congruent with a more recent observational study that not only failed to show harm in transfusion, but showed greater survival in a propensity-matched analysis [25]. An updated randomized trial would be the only means to test this hypothesis adequately [3]. Another important factor could be the issue of timing. ED transfusion may serve to reverse early tissue hypoperfusion and mitigate organ failure. This is supported by a lower incidence of RRT in the transfusion group. ED transfusion may also reduce complications by limiting overall transfusion requirements if it promotes hemostasis and tissue perfusion earlier. In the current study, during the first 24 h after ED admission, cases were transfused PRBC less frequently compared to controls, which may have served to limit the dose-response effect that was observed in patients transfused in both the ED and after admission.

This study has several important limitations. Our analysis did not include all patients transfused in the ED, and was restricted to the number needed based upon the sample size calculation. The results, especially descriptive data regarding transfusion practices, could have been different had the entire transfused sample been included. This highlights the need for further observational and epidemiological data in this domain. The study cohort was not restricted to ICU patients, and our event rate for complications could have been higher had we limited the analysis to ICU admissions. However, our current approach better describes the majority of ED transfusions and is therefore more generalizable. Patients with a hemoglobin <7 g/dl were also excluded. As there is less controversy regarding the risk: benefit of transfusion in this cohort, we wanted to restrict the analysis to patients in whom a transfusion could have potentially been avoided. While we excluded patients with traumatic hemorrhage, those with hemorrhage (potentially major) from a gastrointestinal source were included. This provides valuable descriptive data, but could have further confounded results by including a patient group with a clear indication for transfusion. The cases and controls were well-matched with respect to the a priori matching strategy. However, the cases were potentially a sicker cohort, as demonstrated by a higher ICU admittance rate and greater fluid administration. However, this should have biased our results toward the primary hypothesis, which was not the case.

## Conclusions

In this case-control study, a significant percentage of ED blood product transfusions were discordant with current guideline recommendations. However, there was no association with ED transfusion and worse clinical outcome.

## Additional file

Additional file 1: Definitions of comorbid conditions. (DOCX 13 kb)

## Abbreviations

aOR: Adjusted odds ratio
ARDS: Acute respiratory distress syndrome
CI: Confidence intervals
ED: Emergency department
FFP: Fresh frozen plasma
ICU: Intensive care unit
INR: International normalized ratio
IQR: Interquartile range
PRBC: Packed red blood cells
RRT: Renal replacement therapy
SD: Standard deviation
STROBE: The Strengthening the Reporting of Observational Studies in Epidemiology
TACO: Transfusion-associated circulatory overload
TRALI: Transfusion-related acute lung injury
