# Supplementary material for: Blood product transfusion in emergency department patients: a case-control study of practice patterns and impact on outcome
Source: Int J Emerg Med. 2017 Feb 2;10:5. doi: 10.1186/s12245-017-0133-z (PMC5289930; doi:10.1186/s12245-017-0133-z)
Supplement: Additional file 1: — Definitions of comorbid conditions. (DOCX 13 kb) [file 12245_2017_133_MOESM1_ESM.docx]

**Additional File 1 Definitions of comorbid conditions**

Diabetes Mellitus: Documentation of clinical history in patient’s medical record; current presentation congruent with diabetes mellitus (e.g. diabetic ketoacidosis).

Cirrhosis: Biopsy proven cirrhosis or medical record history suggestive of cirrhosis (ascites, coagulopathy, nodular liver on CT or ultrasound).

Heart failure: Clinical diagnosis on current presentation or history of heart failure in the medical record; includes systolic and diastolic heart failure.

Dialysis/end stage renal disease: Current use of peritoneal dialysis or hemodialysis as an outpatient.

Malignancy: Active or history of; no requirement for history of or current radiation or chemotherapy.

COPD: Not fully reversible airflow limitation; FEV1 <80% + FEV1/FVC <70%; history of COPD in patient’s medical record.

Immunosuppression: Therapy with immunosuppressants, chemotherapy, radiation, long term/recent high dose steroids, active leukemia, lymphoma, or acquired immunodeficiency syndrome (AIDS).

Alcohol abuse: Known diagnosis of chronic alcoholism; previous admission for alcohol detoxification or withdrawal; daily consumption of >14 drinks/week or > 5 binges.

Emergency Surgery: The receipt of surgery within 24 hours of ED presentation.
